# The Effect of Gabapentin on the Efficiency of a Desensitization–Counter-Conditioning Claw-Trimming Protocol for Cats with Healthcare Phobias: A Double-Blind, Placebo-Controlled Crossover Trial

**DOI:** 10.3390/ani15091326

**Published:** 2025-05-03

**Authors:** Lua Raucourt, Sylvia Masson

**Affiliations:** 1 Oniris—École Nationale Vétérinaire, Agroalimentaire et de L’alimentation, 44300 Nantes, France; lua.raucourt@hotmail.com; 2 Clinique Vétérinaire de la Tivollière, 38565 Voreppe, France; 3 No Ledge Research, 38340 Voreppe, France

**Keywords:** gabapentin, healthcare phobia, desensitization–counter-conditioning, fear management, learning protocol, cat welfare

## Abstract

Feline healthcare phobia is a major challenge in veterinary practice, limiting medical care and increasing stress for cats, owners, and veterinarians. Traditional desensitization–counter-conditioning (DS-CC) protocols aim to reduce fear responses, but their success is limited in sensitized cats. This study evaluates whether gabapentin can improve the efficiency of DS-CC training in healthcare phobia in cats. Forty-two sensitized cats participated in a claw-trimming learning protocol. Using a double-blind, placebo-controlled crossover trial, the cats followed 10 training sessions: the first 5 under one product and the last 5 under the other one. Their progression through the learning steps was monitored. The results indicate that gabapentin significantly accelerated the cats’ ability to complete DS-CC steps, suggesting a positive effect on the learning process. While some side effects, such as mild sedation and ataxia, were observed in 42% of the cats, they were transient and did not hinder participation. These findings suggest that gabapentin should be added when engaging patients in DS-CC protocols in order to lower the time needed to achieve therapy, which will improve the overall welfare of our patients. Further studies are needed to confirm these results in other types of therapies and in a clinical setting.

## Simple Summary

Many cats experience extreme fear during veterinary visits, leading to a healthcare phobia. This fear can worsen over time, making handling cats difficult for veterinarians and stressful for both the animal and its owner. Traditional behavioral therapies, such as desensitization and counter-conditioning, are often challenging to implement because highly fearful cats struggle to learn new responses. This study investigates whether gabapentin, a medication known for its anxiolytic effects, can improve the success of a desensitization–counter-conditioning protocol in cats with a healthcare phobia. A group of 42 sensitized cats was recruited, and their progress in a claw-trimming training program was assessed using a double-blind crossover trial of gabapentin versus a placebo. The results show that gabapentin significantly increased the number of training steps completed, facilitating engagement in training sessions. The findings suggest that gabapentin not only improves compliance during examination but also plays a major role in enhancing behavioral therapy for fearful cats, potentially improving veterinary care and animal welfare.

## 1. Introduction

Veterinary visits are a major source of stress for cats, often leading to the development of a healthcare phobia, a behavioral disorder characterized by extreme fear responses to medical procedures [1]. Fear is a natural and adaptive response that enhances survival by promoting the avoidance of potential threats [2]. However, when excessive, fear can become maladaptive, leading to pathological states such as phobias [3]. In veterinary settings, fear reactions in cats result in resistance to handling, a display of aggressive behaviors, and complete immobility due to stress-induced inhibition, all of which compromise their ability to receive medical care [4,5]. This leads to under-medicalization, as owners may avoid veterinary visits due to the distress experienced by their pet [6], ultimately reducing the quality of preventive and curative healthcare [7,8].

To mitigate healthcare phobia in cats, behavioral therapies such as desensitization-counter-conditioning (DS-CC) are commonly recommended [1]. DS-CC involves gradually exposing the cat to a feared stimulus of low intensity while pairing it with appetitive stimuli, thereby reshaping its emotional response [9]. Although effective in many cases, DS-CC protocols have limitations: they are time-consuming, require owner commitment, and often fail when fear levels are too high, preventing effective learning, especially for highly sensitized cats [10]. Sensitization is a process where repeated exposure to a stressful stimulus increases the intensity of fear responses rather than reducing them [1]. The cat is both a prey and a predator species [6], with well-developed hunting instincts, but also vulnerable to other predators. Prey species are thought to be particularly sensitive to danger cues as an adaptive response to predation pressure. While this has been demonstrated in some invertebrate models [11], further research is needed to determine whether a similar mechanism applies to mammals such as cats. However, given the cat’s dual status, its sensitization to stimuli may occur more quickly than it does in other species.

Once a cat becomes highly sensitized to aversive stimuli, it typically exhibits avoidance or escape behaviors (such as hiding, struggling, or biting) to prevent or interrupt the unpleasant experience. From an operant conditioning perspective, these behaviors are negatively reinforced: each successful avoidance or escape provides immediate relief from the perceived threat, thereby increasing the likelihood that the cat will use the same strategy in future similar contexts. This reinforcement cycle contributes to the persistence and escalation of healthcare phobias [8].

The neurophysiological mechanisms underlying fear in cats involve a network of brain structures, including the amygdala, the periaqueductal gray, and the hypothalamus, which coordinate the behavioral, autonomic, and hormonal responses to perceived threats [12]. When a cat perceives a threat, the amygdala sends signals to the periaqueductal gray, triggering fight, flight, or freeze behaviors. Simultaneously, it activates the hypothalamic-pituitary–adrenal axis, resulting in the release of cortisol and adrenaline, which sustain the stress response [11]. In non-phobic animals, the prefrontal cortex can regulate amygdala activity, preventing excessive fear reactions. If sensitization leads to an inappropriate fear response in objectively non-threatening situations, it indicates a dysregulation of the fear system [13]. This is characterized by a failure of the prefrontal cortex to inhibit the amygdala’s reaction or by an overactivation of the amygdala itself, leading to persistent and exaggerated fear responses [14]. This dysregulation inhibits cognitive flexibility, reducing the animal’s ability to learn from new non-fearful experiences and reinforcing avoidance behaviors, which further complicates DS-CC protocols [15,16].

Given the limitations of behavioral therapy alone, pharmacological interventions such as gabapentin, a drug known for its anxiolytic and analgesic properties [17], are increasingly being considered as adjunct treatments to facilitate learning in healthcare-phobic cats. Gabapentin is a structural analog of gamma-aminobutyric acid (GABA) that exerts its effects by binding to the α2δ subunit of voltage-gated calcium channels [18,19]. This action reduces calcium influx at presynaptic terminals, which in turn decreases the release of excitatory neurotransmitters, including glutamate. Although this mechanism is not specific to the amygdala, the amygdala plays a central role in the neural circuitry of fear. By reducing excitatory input to the amygdala and associated structures, gabapentin may help attenuate amygdala hyperreactivity, thereby modulating fear responses.

Gabapentin exhibits high bioavailability (90%) with a half-life of 4 h [20]. Its elimination is strictly renal, requiring dose reduction in cases of renal insufficiency [21]. By reducing calcium influx in presynaptic neurons, gabapentin decreases the release of excitatory neurotransmitters, particularly glutamate, thereby dampening excessive neural excitability [22]. This modulation helps regulate fear responses by limiting amygdala hyperactivity, thereby allowing the prefrontal cortex to exert greater control over emotional reactions, which may improve an animal’s ability to engage in DS-CC training without being overwhelmed by stress responses.

Gabapentin has been shown to reduce acute stress during veterinary visits. For example, it has been proven that the oral administration of 100 mg gabapentin 90 min before reduces signs of stress during transportation [23] and physical examination [24]. It reduces stress levels in cats during short-term postoperative hospitalization [25]. A single 100 or 200 mg oral dose of gabapentin administered by owners 2 h before a veterinary visit can improve the compliance of healthy cats with a history of fear-induced aggressive behavior during a physical examination [26]. A recent study shows that gabapentin is effective in accelerating desensitization protocols in cats with human sensitivity and interspecies social phobia [27]. But gabapentin’s role in enhancing behavioral learning in healthcare-phobic cats remains unproven. By reducing the intensity of fear responses, gabapentin may help healthcare-phobic cats remain cognitively engaged in DS-CC protocols.

This study aims to evaluate whether gabapentin improves the efficiency of a DS-CC protocol in cats with healthcare phobias by facilitating their progression through a structured learning process. By assessing the ability of cats to complete training steps with and without gabapentin, this research aims to provide new insights into the role of pharmacological support in behavioral therapy. The findings could contribute to optimizing treatment strategies for feline phobia, ultimately improving both veterinary care and animal welfare.

## 2. Materials and Methods

### 2.1. Recruitment Protocol

Recruitment was conducted through social media announcements targeting veterinary students, word-of-mouth for private owners, and direct visits to the animal shelter. The shelter’s veterinary assistant pre-selected cats that matched the inclusion criteria. Owners who suspected their cats were fearful based on previous consultations were encouraged to participate.

To ensure homogeneity in the study population, the following inclusion criteria were applied:

- Neutered cats, to avoid hormonal influences on behavior;
- Aged between 6 months and 8 years
- Clinically healthy, confirmed through a clinical examination (see below);
- Fear score ≥ 3 on a 5-point scale (Table 1), assessed during the clinical examination. Difficulty in or impossibility of claw-trimming at home without significant restraint (owner-reported).

The exclusion criteria included the following:

- Aggressive behavior toward humans, defined as a cat that bit the veterinarian during handling;
- Medical conditions that could interfere with the study, including chronic pain, neurological disorders, and suspected renal insufficiency (cats over 8 years were excluded to minimize this risk);

Each candidate cat underwent a standardized veterinary examination to assess both general health and fear responses according to a fear score scale detailed in Table 1. The examination consisted of seven sequential steps, progressing from least (visual inspection of the coat) to most invasive (rectal temperature measurement).

The fear score was quantified using a 5-point scale (Table 1), based on behavioral responses during the examination. Only cats scoring 3 or above were included in the study, ensuring a population already sensitized to veterinary handling.

The examination was conducted without restraint beyond a gentle hand on the cat’s back. If the cat displayed signs of fear corresponding to a score ≥ 3, the examination was paused, and the last completed step of the clinical examination was recorded. The examination was then resumed to complete the health assessment. Cats exceeding a fear score of 2 were considered sensitized and included in the study. Cats displaying extreme aggression (defined as biting during handling) were excluded for safety reasons.

Following recruitment, owners scheduled a first home visit, during which inclusion and exclusion criteria were checked, the veterinary examination was conducted (including fear score assessment), and a behavioral questionnaire was completed to assess suspected comorbidities and the cat’s prior experiences with veterinary visits.

A total of 42 cats were recruited for the study following this procedure.

### 2.2. Study Design

**Figure 1:**
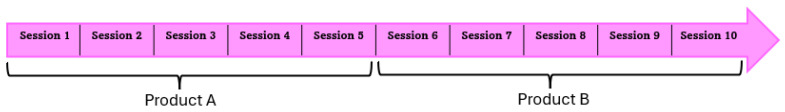
Overview of the crossover study design. *Each cat received gabapentin or a placebo for five consecutive training sessions, followed by the alternate product for five sessions. The order of administration (AB or BA) was randomized.*

This study followed a double-blind crossover design over a period of 10 consecutive training sessions performed within 12 days (5 training days, 2 days without training, and another 5 training days). Each cat was randomly assigned to receive either gabapentin (100 mg/cat) or a placebo for the first five sessions, followed by the alternate treatment for the remaining five sessions, as shown in Figure 1.

To ensure the blinded aspect of the protocol, gabapentin and the placebo were ordered from the pharmacy, and we requested to receive products labeled A and B, unknown to everyone except the pharmacy until the end of the clinical trials. The placebo consisted of milk protein powder, and the capsules were visually indistinguishable.

The order of administration (gabapentin first or placebo first) was randomized (by alternating AB or BA in the order of cat recruitment) to balance out potential order effects.

The training sessions took place at home or in the shelter, as this setting minimizes stress and facilitates learning [30].

**Figure 2:**
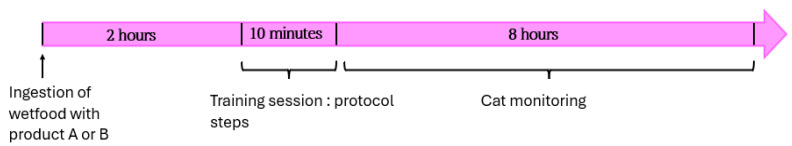
Overview of a single training session. *Each session lasted 10 min and included progressive steps from the desensitization–counter-conditioning (DS-CC) protocol. Product ingestion, behavior, and side effects were recorded.*

Gabapentin or the placebo was administered orally 2 h before each training session, mixed with wet food. Owners were instructed to offer the food spontaneously and not force administration. If the cat refused to eat, this was recorded before the session. Even if the cat completely refused to eat, the session was conducted without the treatment, but data were still collected. A summary of a single training session is presented in Figure 2.

During and after each session, the cat was monitored. Any effects observed by the veterinarian during the session were noted, and after the session, a message was sent by the owner or shelter to report any additional behavioral changes. 

### 2.3. Training Protocol

Before the main study began, the training protocol was tested on three healthy cats without a healthcare phobia. This preliminary phase aimed to achieve two objectives: first, to allow the experimental veterinarian to familiarize herself with the protocol and ensure standardized execution, and second, to confirm that healthy cats could successfully complete all training steps within the 10-session timeframe. Each of these three cats underwent the full protocol and was able to complete all steps within under 8 sessions, demonstrating the feasibility of the training design and establishing that any delays in progression observed in the study cats would likely be attributable to a healthcare phobia rather than limitations of the protocol itself. Sessions lasted 10 min and were conducted daily for two weeks, excluding weekends.

A desensitization–counter-conditioning (DS-CC) protocol was designed to train cats to tolerate claw-trimming. The CC component was designed as an operant counter-conditioning procedure. The aim was to encourage the display of calm, alternative behaviors in the presence of stimuli already associated with fear. Appetitive stimuli (food rewards) were provided contingent upon the performance of specific target behaviors during each step of the protocol, which was administered by an unfamiliar person (the veterinarian), reinforcing the aversive context of the training environment [31].

The protocol consisted of 8 progressive steps, as shown in Table 2, starting from remaining on the table to allowing claw extension without any tool to an actual trimming action, structured to ensure gradual exposure to handling while reinforcing calm behavior. None of the recruited cats had ever practiced any steps of the protocol before.

Advancement to the next step required the cat to successfully complete the current step three consecutive times within the same session, without displaying signs of stress or avoidance. If the success criterion was not met, the session continued at the same step without regression or forced repetition of earlier steps. This flexible approach allowed each cat to progress at its own pace while minimizing stress and maintaining positive associations.

Cats were positively reinforced with food rewards when they successfully completed a step. A consistent “click” sound was made with the mouth immediately after the desired behavior, followed by placement of the treat on the ground in front of the cat to allow voluntary consumption.

Palatability was individually assessed during the recruitment session. While most cats responded positively to Catisfactions^®^, alternative treats such as wet food sachets or canned tuna were used in five cases to accommodate individual preferences and ensure effective reinforcement.

The highest step successfully completed during each session was recorded immediately after the session by the veterinarian on an individual tracking sheet.

### 2.4. Behavioral Analysis

Behavioral analysis focused on training progression (ranging from 0 to 8), ingestion monitoring, and potential side effects, as explained in Table 3. The primary outcome was the highest training step successfully completed per session. Progression through the desensitization–counter-conditioning (DS-CC) protocol was recorded, with the difference between the first and last completed steps under each treatment condition (gabapentin or placebo) used to quantify learning efficiency. Additionally, the total progress over the 10 sessions was reported for each cat.

Ingestion monitoring was recorded in each session by noting the quantity of food consumed. Ingestion was recorded as “0” if the cat did not eat any of the provided portion and “100%” if the cat ate it all. Fort the rest, it was noted as “less than half consumed” or “more than half”. Sessions during which cats consumed less than 50% of their allocated product were analyzed separately to assess the dose-dependent effects on training progression.

Additionally, side effects were monitored in every session by owners and the experimental veterinarian, with reported symptoms including sedation, ataxia, increased appetite, and general fatigue.

### 2.5. Statistical Analysis

Statistical analysis was performed using R software version 4.4.2. Shapiro–Wilk tests were used to verify data normality, leading to the choice of non-parametric methods.

Wilcoxon’s signed-rank tests were used to compare quantitative paired data such as progress between the two treatments (gabapentin or placebo), as both were administered to each cat. To account for multiple factors and random effects, a linear mixed-effects model analyzed the influence of treatment order and cat category on progress under the placebo and gabapentin. Spearman’s correlation tested the relationship between observed effects and the administered dose.

A significance level of 0.05 was considered for all tests.

### 2.6. Ethical Considerations

The study was conducted in accordance with ethical guidelines for animal research. The owners provided written consent, and all procedures were non-invasive. The study received ethical approval from the Oniris’ Ethics Committee for clinical and epidemiological Veterinary Research (CERVO) under approval code CERVO-2024-10-V.

## 3. Results

### 3.1. Cohort Study

#### Population Characteristics

A total of 42 cats were recruited for the study, including 20 from an animal shelter and 22 from private owners. The recruited population consisted of 55% females, with ages ranging from 9 months to 8 years (mean = 3.8 years, variance = 4.8). The weight of the cats varied from 2.8 kg to 6.2 kg (mean = 4.2 kg, variance = 0.7).

During the study, seven cats were withdrawn for different reasons:

- Three were adopted from the shelter and hence could not complete the full protocol;
- Two were unavailable for more than one training session;
- One experienced significant fatigue after the administration of product B and the owner chose to retire the cat from the protocol;
- One displayed aggression during the sixth session (first session under placebo after five sessions under gabapentin).

As a result, 35 cats completed the full protocol of 10 training sessions. Among them,

- Twenty-two cats fully ingested both products;
- Eleven cats partially ingested gabapentin but fully ingested the placebo;
- Two cats refused to ingest gabapentin entirely and were excluded from statistical analysis.

### 3.2. Ingestion Rates

Among the 35 included cats, 37% (n = 13) did not fully ingest gabapentin, whereas 100% of the cats ingested the placebo without difficulty, as shown in Table 4.

Of the cats that received the placebo first, 69% ingested gabapentin entirely when offered it during the second week, compared to only 54% of those who received gabapentin first.

### 3.3. Adverse Effects

Adverse effects were reported in 42% of the cats, as detailed in Table 5. A cat could experience one or more side effects, such as mild ataxia and increased appetite, for example.

### 3.4. Training Progression

#### 3.4.1. Effect of Gabapentin on Learning Progress

##### Among the 22 Cats That Ingested Both Products

**Figure 3:**
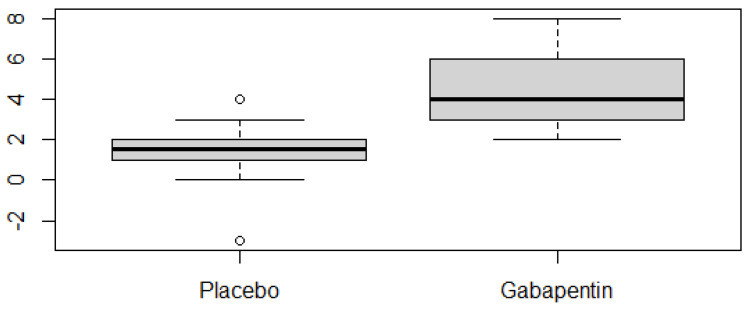
Boxplot showing the number of DS-CC training steps progressed to by each cat during the five-session period under placebo and gabapentin (n = 22 cats with full ingestion of both treatments). Progression was calculated independently for each condition by subtracting the starting step from the final step reached at the end of the five sessions.

A comparison of the two conditions (placebo vs. gabapentin) among the 22 cats that ingested both products clearly showed significantly faster learning progress with gabapentin (Wilcoxon’s test, W = 43.5, *p* = 2.37 × 10^−6^), as shown in Figure 3. The effect size (d = 0.869) confirmed a strong effect of the drug on learning speed.

##### Among the 11 Cats Who Partially Ingested Gabapentin

To determine whether gabapentin still facilitated learning when taken at a reduced dose, we compared the progress of each cat between sessions where they consumed more than half of the prescribed dose (or the full dose) of gabapentin and sessions where they consumed less than 50% of the dose of gabapentin, no gabapentin at all, or the placebo. The results showed that the number of training steps completed was significantly higher in sessions where cats ingested at least half of the intended gabapentin dose compared to sessions where they did not ingest it (Wilcoxon’s paired test, *p* < 0.005, W = 66, effect size d = 0.79). This suggests that a dose of 50 mg/cat is sufficient to accelerate learning, although the effect was less pronounced than with the full 100 mg/cat dose (*p* < 2.38 × 10^−6^).

Conversely, when analyzing sessions where cats ingested less than 50% of the intended dose, no significant difference was found in progress compared to sessions where gabapentin was not ingested (Wilcoxon’s paired test, *p* = 0.201, W = 9, effect size d = 0.35). This indicates that when a cat consumes less than 50 mg, the drug no longer has a measurable effect on learning acceleration. Thus, gabapentin only facilitates learning when administered at a minimum of half the recommended dose (≥50 mg/cat), while lower doses do not significantly impact training progression.

##### Among the Two Cats Who Did Not Ingest Gabapentin

**Figure 4:**
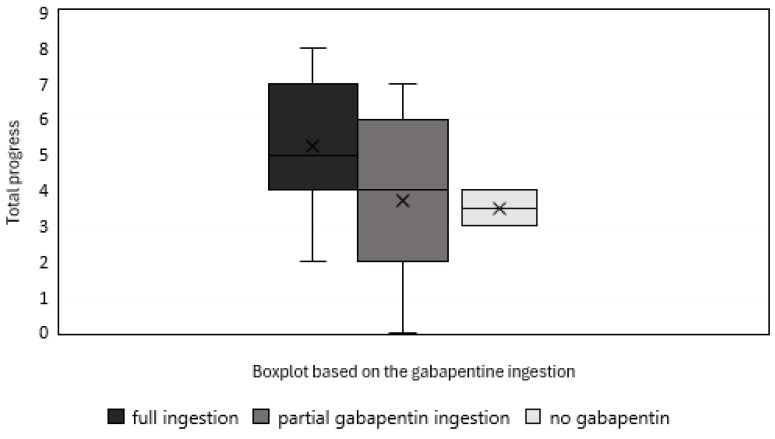
Boxplot illustrating the total progress of cats that fully ingested both products (n = 22), partially ingested gabapentin (n = 11), and refused gabapentin entirely (n = 2).

The boxplot shown in Figure 4 illustrates the total progress of cats by the end of the 10-session training, categorized based on their gabapentin ingestion. Cats that fully consumed both products (n = 22) showed the highest median progress, while those that only partially ingested gabapentin (n = 11) had a slightly lower median but a wider variability. The two cats that refused gabapentin entirely (n = 2) demonstrated the least progress; however, given the very small sample size, this result should be interpreted with caution. Despite having only five medicated sessions for those that ingested gabapentin, the trend suggests that higher gabapentin intake is associated with greater overall progress, reinforcing its potential role in facilitating learning.

#### 3.4.2. Influence of Initial Product Given

Regression analysis of the 22 cats that ingested both products showed that gabapentin had a significant effect regardless of the administration order, as shown in Table 6. Indeed, there was no significant interaction between product efficacy and administration order (*p* = 0.2192, R^2^ = 0.458), indicating that the effect of gabapentin was independent of whether it was given first or second.

#### 3.4.3. Effect of Suspected Comorbidities

Regression analysis of the 22 cats that ingested both products showed no significant difference in progress between cats suspected of having behavioral comorbidities and those with only a healthcare phobia (*p* = 0.5953, R^2^ = 0.431).

#### 3.4.4. Dose–Effect Correlation

Spearman correlation tests were performed to assess the relationship between gabapentin dose (mg/kg) and learning progress and showed that no significant correlation was found in cats that fully ingested gabapentin (rho = 0.184, *p* = 0.4124), nor in those who ingested it partially (rho = −0.442, *p* = 0.1736).

## 4. Discussion

This study is the first to demonstrate that gabapentin facilitates learning in a desensitization–counter-conditioning claw-trimming protocol for cats with healthcare phobias. The results show that cats receiving gabapentin progressed significantly faster through the training steps than those in the placebo group, reaching higher protocol steps in fewer sessions. This suggests that gabapentin enhances cognitive flexibility or reduces fear, allowing learning when faced with fear-inducing stimuli.

Although the results are promising, some methodological considerations must be acknowledged. No interobserver agreement assessment or formal procedural fidelity measurement was conducted. Instead, we relied on a binary step-based scoring system, and all training sessions were conducted by the same trained veterinarian, following a standardized sequence practiced beforehand on non-fearful cats. This design choice aimed to minimize variability and ensure internal consistency. Nevertheless, the absence of formal independent validation represents a limitation that should be addressed in future studies. These findings align with previous research highlighting gabapentin’s ability to reduce acute stress during veterinary handling [23,24,26]. However, unlike prior studies that primarily focused on its short-term effects, potentially attributable to sedation, this study emphasizes its role in improving behavioral training outcomes in a therapeutic context. This distinction is crucial, as it suggests that gabapentin is not merely a chemical restraint but an essential aid in behavioral modification protocols.

The order of administration (gabapentin first or placebo first) had no significant impact on learning progression, indicating that the observed effects were not simply due to a practice effect but rather to a genuine facilitation of learning.

Additionally, suspected behavioral comorbidities did not significantly alter learning progression in the specific context of claw-trimming. Future studies could refine participant selection by including only cats evaluated by a behavioral specialist rather than relying on owner questionnaires to validate or invalidate this absence or correlation. This would provide a more precise understanding of how pre-existing behavioral sensitivities influence DS-CC training success.

Despite its effectiveness, gabapentin was not fully ingested by 37% of the cats, which may have led to an underestimation of its true potential. However, even in cases of partial ingestion, learning progression remained superior to that of non-treated cats. Future studies should explore strategies to increase ingestion rates, such as implementing a habituation phase before administration. In our protocol, cats were allowed to freely accept or refuse the medicated wet food, and this proportion of 37% that refused it would probably be reduced if specific attention was paid to creating a food ritual a few days before presenting the food with gabapentin.

Gabapentin was generally well tolerated, but adverse effects were reported in 33% of cases. The most frequently observed signs were ataxia, sedation, or fatigue [32,33] and increased appetite [34] in proportions similar to previous studies. One 3 kg cat experienced marked ataxia for five hours, which was resolved after reducing the dose by half. Another cat developed severe fatigue and dysorexia lasting three days, requiring an emergency consultation. This cat was diagnosed with febrile syndrome (39.5 °C), thrombocytopenia, and leukopenia. These symptoms were resolved with antibiotic and appetite stimulant treatment, and the attending veterinarians did not attribute the episode to gabapentin. Nevertheless, the cat was removed from the study. Although a causal link was not established, this highlights the importance of monitoring individual tolerance and adjusting the dose when necessary. The lack of a clear dose–response relationship further supports the idea that individual pharmacodynamic variability may influence the efficacy and safety profile of gabapentin in cats.

However, the causal link between gabapentin ingestion and the symptoms presented by the cat was not clearly established by the veterinarian that took care of this cat, but this underscores the importance of monitoring individual tolerance and considering dose adjustments in certain cases. The lack of a dose–effect correlation further supports the idea that gabapentin’s benefits in learning facilitation are not strictly dose-dependent but rather influenced by individual variability in drug metabolism.

This study focused exclusively on claw-trimming, a common yet challenging veterinary procedure. This choice was strategic, as it represents an ethically acceptable, moderately difficult procedure that many cats struggle to tolerate. While these findings support gabapentin’s efficacy in this context, they cannot yet be generalized to other veterinary procedures. Although healthcare-related fear and nail-trimming-specific fear may differ conceptually, all cats included in this study had demonstrated claw-trimming avoidance at home, as reported by their owners. This was part of the inclusion criteria and ensured that the selected population did exhibit behavior consistent with claw-trimming-related fear or aversion. Thus, while the task may also reflect general handling sensitivity, it remains a valid and ecologically relevant model of healthcare-associated fear in cats.

Although the sample size allowed for statistically significant results, a larger cohort would strengthen the robustness of these findings.

Another limitation of this study is that the difficulty of the training steps was not necessarily equivalent for all cats. Some individuals may have found certain steps more challenging than others, potentially influencing overall progression. A descriptive overview of the average number of sessions required per step is provided in Supplementary Figure S1. However, this effect was mitigated by the crossover design, ensuring that each cat was exposed to both conditions (gabapentin and placebo), allowing for a more reliable assessment of gabapentin’s impact on learning. Future studies could refine training steps to ensure greater consistency in difficulty levels across subjects.

Moreover, the short-term nature of this study is a limitation. While it demonstrated an acceleration in learning, it did not evaluate long-term retention of the acquired behaviors. Follow-up studies are needed to assess whether gabapentin provides lasting benefits in DS-CC training.

The study was conducted in a home setting, where cats are naturally less stressed. Investigating whether the effects of gabapentin persist in a clinical environment would provide valuable insights into its real-world applicability. Future research should also determine whether the dose used in this study remains effective in a clinical setting or whether adjustments are needed to maintain efficacy under higher baseline stress.

## 5. Conclusions

This study demonstrates that gabapentin significantly accelerates the progression of cats through the DS-CC protocol for claw-trimming. For some cats, it may actually even render the DS-CC protocol feasible. While gabapentin is already known to facilitate handling in veterinary contexts, these results suggest that its effects extend beyond simple restraint, potentially enhancing learning in healthcare-phobic cats. Furthermore, the observed acceleration in learning was independent of dose, administration order, and suspected behavioral comorbidities, reinforcing the robustness of the findings.

Despite these promising results, several limitations must be considered, including variability in gabapentin ingestion, only one claw-trimming exercise being measured, and the study’s home-based setting. Addressing these limitations in future studies would help refine its therapeutic potential.

This study supports the early integration of gabapentin into behavioral therapy protocols for healthcare-phobic cats, shifting the perception of medication use in veterinary behavioral medicine from a last resort to a proactive therapeutic tool. Enhancing veterinarians’ awareness of early fear identification could greatly improve feline welfare and increase owner compliance with veterinary care. By administering gabapentin early, the development of healthcare phobias could be prevented, making treatment more effective than managing already sensitized cats, which is significantly more challenging.

## Figures and Tables

**Table 1:** Numerical rating scale to assess the fear score during the clinical examination. Modified from Mills et al. [28], van Haaften et al. [23], and Lamminen et al. [29].

Score	Description
1	Clinical examination could be easily performed without resistance or with insignificant resistance (no restraint needed). Cat was compliant, not frozen, and did not express signs of fear
2	Minor resistance. Clinical examination could be performed with the technician minimally restraining the cat by placing a hand on the back.
3	Moderate resistance. Clinical examination could be performed with the veterinary technician using physical restraint involving stabilizing the cat and holding it in place. Cat expressed moderate signs of stress with a moderately tense body.
4	Strong resistance or freezing. Clinical examination could be performed with the veterinary technician more tightly restraining the cat (physically wrapping of scruffing cat).
5	Extremely strong resistance. The cat responded to the clinical examination with avoidant and/or defensive behavior to an extent that completing the examination required sedation.

**Table 2:** Eight progressive steps of the DS-CC protocol.

Step	Description
1	Remaining on the table
2	Sitting down
3	Giving a paw without attempting to withdraw it
4	Allowing the paw to be held for 5 s while staying still
5	Allowing claw extension without any tool present
6	Allowing the claws to be touched with the nail clipper (without cutting)
7	Remaining still while a single claw is trimmed
8	Remaining still while all claws on one paw are trimmed

**Table 3:** Variable, range, and explanation.

Variable	Range/Value	Meaning
Progress under A (i.e., change in training step)	0 to 8	That is, (The protocol step at which the cat stopped at the end of the 5 sessions with product A) − (The protocol step at which the cat started before product A)
Progress under B	0 to 8	That is, (The step at which the cat stopped at the end of the 5 sessions with product B) − (The step at which the cat started before product B)
Progress under A and B	0 to 8	Total progress made by the cat over the 10 sessions
Side effects	Open opinion	After each session, the cat was monitored, and any behavioral changes were reported by the owner or shelter
Ingestion	“0”; “less than half consumed”; more than half consumed”; “100%”	“0” if the cat did not eat any of the provided portion and “100%” if the cat ate it all. For the rest, it was noted as “less than half consumed” or “more than half consumed”.
Category	1 or 2	According to the behavioral questionnaire, Category 1 = no behavioral comorbidity to healthcare phobia suspected;Category 2 = behavioral comorbidity suspected

**Table 4:** Comparison of ingestion rates.

Product	Full Ingestion	Partial Ingestion	Refusal
Placebo	35/35 (100%)	0%	0%
Gabapentin	22/35 (63%)	11/35 (31%)	2/35 (6%)

**Table 5:** Reported adverse effects of gabapentin at 100 mg/cat (n = 33 cats in sessions during which they received 100 mg of gabapentin).

Adverse Effect	Number of Cats	Proportion (%)
Mild to moderate ataxia (<8 h)	8	24%
Severe ataxia (>8 h)	1	3%
Fatigue (<8 h)	11	33%
Intense fatigue (>8 h) and dysorexia	1	3%
Increased appetite	9	27%
*No adverse effect at all*	*19*	*58%*

**Table 6:** Regression analysis of progress as a function of the product and order of administration.

Fixed Effect	Estimate	Std. Error	DF	t-Value	*p*-Value
Intercept	1.846	0.445	20	4.15	0.0005
Gabapentin	2.308	0.629	20	3.67	0.0015
Order (gabapentin first)	−1.068	0.696	20	−1.54	0.1402
Gabapentin × Order	1.248	0.984	20	1.27	**0.2192**

## Data Availability

The raw data supporting the conclusions of this article will be made available by the authors upon request.

## Supplementary Materials

The following supporting information can be downloaded at https://www.mdpi.com/article/10.3390/ani15091326/s1, Figure S1. Mean number of training sessions required to successfully complete each of the eight protocol steps (n = 22 cats). Error bars represent 95% confidence intervals. A repeated-measures ANOVA showed a marginally non-significant overall effect of step (*p* = 0.087). Step 7 showed the highest average difficulty, but no individual comparison reached significance after Bonferroni correction.

## Abbreviations

The following abbreviations are used in this manuscript:

DS-CC	Desensitization counter-conditioning
SSRIs	Selective serotonin reuptake inhibitors
CERVO	Oniris’ Ethics Committee for clinical and epidemiological Veterinary Research
