# Supplementary material for: The Effect of Gabapentin on the Efficiency of a Desensitization–Counter-Conditioning Claw-Trimming Protocol for Cats with Healthcare Phobias: A Double-Blind, Placebo-Controlled Crossover Trial
Source: Animals (Basel). 2025 May 3;15(9):1326. doi: 10.3390/ani15091326 (PMC12070960; doi:10.3390/ani15091326)
Supplement: Supplementary file 1 [file animals-15-01326-s001.zip › animals-3584280-supplementary.pdf]

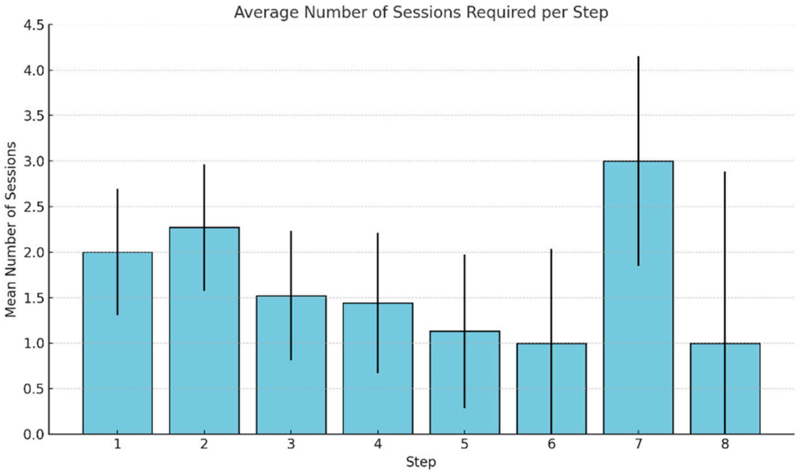

Supplementary Figure S1. Mean number of training sessions required to successfully complete each of the eight protocol steps (n = 22 cats). Error bars represent 95% confidence intervals. A repeated measures ANOVA showed a marginally non-significant overall effect of step ($p = 0.087$). Step 7 showed the highest average difficulty, but no individual comparison reached significance after Bonferroni correction.
